# Valorization of Agro-Industrial Wastes as Organic Amendments to Reduce Herbicide Leaching into Soil

**DOI:** 10.3390/jox15040100

**Published:** 2025-06-30

**Authors:** Gabriel Pérez-Lucas, Andrea Martínez-Zapata, Simón Navarro

**Affiliations:** Department of Agricultural Chemistry, Geology and Pedology, School of Chemistry, University of Murcia, Campus Universitario de Espinardo, E-30100 Murcia, Spain; andrea.martinezz@um.es (A.M.-Z.); snavarro@um.es (S.N.)

**Keywords:** herbicides, downward movement, leaching indexes, soil half-lives, organic amendments

## Abstract

High levels of pesticide use are associated with intensive crop production. Pesticides are increasingly prevalent in surface and groundwater, which is a major environmental concern. Various methods have been proposed to improve the retention and/or degradation of pesticides in soils. These methods are mainly based on soil adaptation with organic wastes to mitigate soil and water pollution. In addition, there has recently been increased interest in assessing the influence of organic waste additions on pesticide movement in soils with low contents of organic matter. Agriculture and related industries generate large amounts of waste each year. Because of their components, they have the great ability to produce high-value products for environmental restoration. This study reports on the influence of four different agro-industrial wastes (orange peel, beer bagasse, grape pomace, and gazpacho waste) used as organic amendments on the leaching of metobromuron and chlorbromuron (phenylurea herbicides) on a silty clay loam soil (gypsic–calcaric regosol) with low organic matter contents from a semiarid area (southeastern Spain). The adsorption, leaching, and dissipation processes of these herbicides were evaluated on a laboratory scale in amended and unamended soils. In addition, the main leaching indices (GUS, LIX, LEACH, M LEACH, LIN, GLI, HI, and ELI) commonly used to assess groundwater protection against pesticide pollution were evaluated. The sorption coefficients (*K*_OC_) increased in the amended soils. Metobromuron was found in leachates in all cases, although a marked reduction was observed in amended soils, while chlorbromuron was mainly retained in soils, especially in the top layer. The disappearance time (DT_50_) for metobromuron and chlorbromuron in soil ranged from 11 to 56 d and 18 to 95 d, respectively. All indices except GLI categorize metobromuron as mobile or very mobile in unamended soil. For chlorbromuron, GUS, LIX, LEACH, MLEACH, and Hornsby classify this compound as a medium-to-high leache, while GLI and ELI classify it as having low mobility. In amended soils, most indices classify metobromuron as transitioning to mobile, while most indices catalog chlorbromuron as immobile/transition.

## 1. Introduction

Human food comes primarily from agriculture. Advances in agricultural technology over the last few decades have led to a qualitative and quantitative leap in food production, driven by the need to feed a continuously growing world population of around 2% per year. The current world population is more than three times higher than it was in the mid-twentieth century and reached 8 billion in mid-November 2022. It is estimated that the population will increase by nearly 2 billion people in the next 30 years, growing from 8.2 billion today to 9.7 billion in 2050 and potentially reaching 10.4 billion by 2080 [1]. However, progress has often come with negative social and environmental consequences. These include water scarcity, soil degradation, pressure on ecosystems, loss of biodiversity, and a decline in fish stocks and forest areas. Other consequences are the pollution of environmental compartments and high levels of greenhouse gas emissions (CO_2_, N_2_O, CH_4_, and F-gases).

For these reasons, achieving sustainable development is one of today’s greatest challenges. Development will be sustainable if a balance is achieved between the factors influencing quality of life through the rational exploitation of resources, thereby satisfying the needs of current societies without compromising those of future ones. Thus, the main objective of sustainable development is to increase food production while making rational use of natural resources.

Agriculture relies heavily on phytosanitary products and other tools to protect plant health and control pests, diseases, and weeds that reduce crop yield. Agricultural losses reduce the global food supply, damage the economy and the environment, and waste the natural resources used in production. Weeds cause 34% of the global agricultural potential loss, compared to 18% and 16% caused by animal pests and pathogens, respectively [2]. Weeds are often more harmful than pests and diseases because they develop quickly. Weeds compete with economic crops for essential resources such as light, water, and soil nutrients, which significantly impact agro-biodiversity, yield, and quality while raising the overall cost of production. The growth and spread of weeds, pathogenic organisms, and insects are affected by abiotic stress factors such as drought and temperature fluctuations. Therefore, climate change could decrease agricultural efficiency by 10–25%, and this decline is expected to intensify over the next 50 years [3,4].

In 2022, the total use of pesticides in agriculture was 3.7 million tons (Mt) of active ingredients (1.95 Mt herbicides, 0.79 Mt fungicides and bactericides, 0.77 Mt insecticides, and 0.2 Mt other pesticides), marking a 13% increase over the past decade and a doubling since 1990. From 1990 to 2022, pesticide use increased in intensity at different rates. The use per cropland area saw a 94% increase, the use per value of agricultural production saw a 5% increase, and the use per person saw a 35% increase [5].

In recent years, the increasing globalization of the market, together with rising temperatures, has created favorable conditions for the movement and establishment of plant pathogens, consequently increasing the risk of serious yield losses [4]. The effects of climate change not only promote the spread of pests but also threaten the survival of beneficial plant and insect species, including pollinators of the most economically and socially important crops. This poses a growing threat to food security and the environment. The ultimate consequences are a decrease in crop production and food obtainability, which may lead to starvation in some areas.

Because of the adverse effects of synthetic pesticides, finding safer, nature-based alternatives is one of the most important goals. These include ways to cultivate plants differently, use biological pest control, engineer plants in a new way, and interfere with how insects breed. The most usual alternative to traditional pesticides is biopesticides.

Although applying herbicides to agricultural soils is an effective way to control weed growth, it has raised environmental concerns due to the low biodegradability and long-term persistence of some herbicides in soil. Therefore, herbicide residues can reach surface and groundwater via runoff and leaching. Normal field applications of certain herbicides can result in the leaching of those chemicals into groundwater under specific conditions. Herbicides can travel from soil to groundwater. They do so through matrix flow. However, in some cases, macropores act as favored flow pathways, causing pollutants to move rapidly through the unsaturated zone [6]. The physicochemical properties of herbicide, soil texture, clay and organic matter (OM) content, and soil permeability play a decisive role in the leaching process [7]. However, the amount of organic carbon (OC) in the soil is the single largest factor influencing the adsorption and mobility of pesticides and, consequently, its disappearance [8]. Thus, one possible mitigation measure to reduce the leaching of pesticides through the soil is to increase the soil organic matter (SOM) content, which will improve soil quality and reduce the amount of pesticides that leach through the soil through agronomic practices, such as incorporating crop residues or animal manure, to increase the sorption of non-ionic pesticides [9]. In this context, sustainable agricultural practices, such as adding organic amendments to soil, are becoming increasingly necessary because of long-term intensive agriculture. This is because it can compromise soil health [10,11].

In recent years, many studies have shown the effects of SOM on the adsorption, degradation, and leaching of pesticides in soil [12,13,14,15,16,17,18,19,20,21,22,23,24,25,26,27,28]. According to Tiryaki and Temur [29], pesticide leaching is a significant concern for compounds that exhibit weak adsorption and prolonged persistence in climates with low temperatures and high rainfall. This is due to the impact of groundwater re-loading, particularly in areas with limited OM content and a sandy soil texture. The *K*_OC_ ratio (soil organic adsorption coefficient), which is usually expressed as log *K*_OC_, is used to evaluate the relative mobility potential of nonionic herbicides in soils. It is described as the concentration of a compound in a sorbed state (adhering to soil particles) and in the solution phase (dissolved in water), bearing in mind the organic carbon content. The *K*_OC_ is a universally accepted measure of the relative mobility of pesticides in soil, which is important for understanding how they move through soil and how they might affect crops. It groups pesticides from immobile to mobile, and it is also used in fugacity models to describe how pesticides are distributed in soil, water, and air.

Over time, the entire agri-food production supply chain has been negatively impacted by waste from the agriculture and food industries [30]. The traditional agricultural production model is circular. Virtually no waste is produced, which is a significant benefit. Residues are usually returned to the soil as compost or used as bedding material in livestock husbandry. They are also used to produce animal protein and manure. Soil conservation, waste reduction, and the reuse and recycling of residues are all ensured by circular agricultural production. However, the ever-growing global population and demand for food and agro-industrial products have led to a shift towards linear agricultural production, which results in the production of substantial amounts of agricultural, agro-industrial, and food waste. Waste management models that are currently in use focus on circular agricultural production and bioeconomic approaches that aim to reduce, reuse, and recycle waste. These approaches view agricultural waste as a valuable resource that can benefit farmers, consumers, and investors in various industries.

The United Nations’ 2030 Agenda for Sustainable Development highlights the importance of ensuring sustainable consumption and production patterns. Particularly, the United Nations recommends circular economy strategies (prevention, reduction, recycling, and reuse) to reduce waste generation [31]. In March 2020, the EC adopted a new Circular Economy Action Plan to this end. The plan is comprehensive, with initiatives that cover the entire product life cycle. Therefore, the food sector has been identified as a key area with significant potential for making the circular economy a reality. In this context, the agriculture sector and related industries produce around 1.3 billion tons of waste annually [32]. Currently, most of this waste is disposed of through incineration or landfilling. This method poses substantial economic, social, and environmental challenges.

For these reasons, developing a sustainable agro-industrial waste management system is necessary. Their composition makes these wastes valuable sources of high-end products. Agro-industrial waste can be categorized as either on-farm or off-farm. On-farm waste includes agricultural residues, such as leaves, seeds, stems, and bunches that are left in the field after harvesting. Off-farm waste includes industrial waste, such as field residues and process residues. Field residues are the husks, roots, and seeds left in a field after harvesting. Process residues are the peels, oil, pomace, grounds, and molasses generated from processing raw products [33]. In addition to uses such as livestock production, renewable energy production, and the extraction of bioactive molecules, among other uses, agro-industrial residues have several benefits for soil health, including preventing or minimizing soil erosion, increasing SOM, maintaining soil OC, improving soil structure, conserving soil moisture, recycling plant nutrients, and maintaining soil organisms, microbial populations, and fertility. Some authors have demonstrated that adding agro-industrial waste to soil significantly reduces pesticide leaching [14,16,20,22,34]. Organic residues that can be used as organic amendments in soil include those from urban activities (e.g., sewage sludge and solid waste), agricultural activities (e.g., crop residues), livestock activities (e.g., manure and slurry), and agro-industrial activities (e.g., wine, juice, beer, olive production, and others).

Two levels exist for assessing pesticide mobility: one in a laboratory setting with disturbed/undisturbed soil columns as described by Katagi [35], and the other in the field using soil lysimeters, which are primarily used to evaluate the transfer of pesticides from the soil surface to groundwater [36]. The two main procedures proposed by the Organization for Economic Cooperation and Development (OECD) [37] and the United States Environmental Protection Agency (US EPA) [38] are often used to assess pesticide transport through the soil on the laboratory scale. Several methods have been proposed to assess pesticide mobility. The most prominent indices available in recent decades are reviewed in the papers by Pérez-Lucas et al. [6] and Araya et al. [39]. Other indices include YASGEP-P, which is a composite of diverse indices created using principal component analysis [40], as well as the experimental leaching index (ELI), which is based on experimental data and the precipitation regime in a detailed area [21]. With this aim, this study reports on the impact of four different agro-industrial wastes (orange peel, beer bagasse, grape pomace, and gazpacho waste) used as soil amendments on the mobility of two commonly used phenylurea herbicides (metobromuron and chlorbromuron) on a silty clay loam soil with low OM contents from a semiarid area (southeastern Spain).

## 2. Materials and Methods

### 2.1. Herbicides, Solvents and Reagents

The analytical standards of the herbicides metobromuron (MB) and chlorbromuron (CB), both with a purity of 99.5%, were purchased from Dr. Ehrenstorfer GmbH (Augsburg, Germany). The main physicochemical characteristics of both herbicides are shown in Table 1. All HPLC-grade solvents (H_2_O and CH_3_CN) and reagents (NaCl and CaCl_2_) with purities greater than 98.5% were obtained from Scharlab (Barcelona, Spain).

### 2.2. Soil and Organic Amendments (Agro-Industrial Wastes) Used

The soil used was a gypsic–calcaric regosol [43] with a silty clay loam texture and bulk density = 0 1.28 g cm^−3^. Soil samples were collected from the top 20 cm of the soil in the Campo de Cartagena (SE, Spain), air-dried, and sieved (2 mm). The main physical chemical properties of the soil are shown in Table 2.

Four different agro-industrial waste products were used as organic amendments: (i) orange peel (OP), obtained during juice production and supplied by AMC Grupo Alimentación (Murcia, Spain); (ii) spent brewers grains (SG), provided by Estrella de Levante Fábrica de Cerveza (Murcia, Spain); (iii) gazpacho waste (GW), supplied by AMC Grupo Alimentación; and (iv) grape pomace (GP) of the Garnacha Tintorera variety from Casa de la Ermita Winery (Jumilla, Spain). The wastes were air-dried, and then placed in an oven at 60–70 °C for two days until they lost all moisture. Finally, the wastes were crushed and sieved to a size of 0.3 mm. Table 3 shows their main physicochemical characteristics. Enough of each amendment was added to each soil sample to increase the soil’s organic matter content to 3%.

### 2.3. Experimental Setup

The batch equilibrium method proposed by OECD [44] was used to determine the adsorption behavior of MB and CB. Five-gram soil samples in triplicate were added to 50 mL centrifuge tubes containing 25 mL of a 0.1 μg mL^−1^ herbicide solution in 0.01-M CaCl_2_. The mixture was shaken until adsorption equilibrium was reached, which took 24 h at a temperature of 20 ± 1 °C. Preliminary experiments showed that equilibrium could be reached after 24 h of contact. Finally, the tubes were subjected to centrifugation (4000 rpm for 10 min), and the resultant fluid was filtered through a 0.22 μm nylon filter. The amount adsorbed (*C*_a_) was established by subtracting the initial concentration in solution (*C*_i_) from the concentration remaining after equilibration (*C*e). The distribution coefficient (*K*_d_) was acquired from the relationship between *C*_a_ and *C*e (*K*_d_ = *C*_a_/*C*e). Finally, the *K*_OC_ was calculated as follows: *K*_OC_ = (*K*_d_ × 100)/% OC.

The OECD procedure [45] was used to conduct experiments to study soil degradation. The evaluation of herbicide persistence involved the preparation of soil samples, with each sample weighing 50 g based on oven-dry weight. These samples were then placed in incubation flasks for further analysis. Then, 250 µL of an acetonitrile/water solution (50/50 *v*/*v*) containing 100 µg mL^−1^ of each herbicide was applied to achieve 0.5 µg g^−1^ of each herbicide and the flasks (n = 3) were incubated in the dark at 21 ± 2 °C, while maintaining a soil moisture level of 30% of field capacity (0.35). Flasks from each treatment were removed after 7, 15, 30, and 60 days. They were stored at 4 °C for no more than three days before analysis. Water losses exceeding 10% were compensated for weekly by adding distilled water. Following the FOCUS work group’s guidelines [46], the dissipation data for the herbicides were fitted to the single first-order (SFO) kinetic model according to the following expression: *C*_t_ = *C*_0_ e^−*k*t^ (ln *C*_t_ = Ln *C*_0_ − *k*t). Here, *C*_0_ is the initial herbicide concentration, *k* is the rate constant (day^−1^), *C*_t_ is the herbicide concentration in the soil at a given time, and t is the time (days) since the herbicides were added.

The OECD guidelines [37] were used as a framework for the study of the leaching behavior. In brief, the downward movement of herbicides was studied in polyethylene terephthalate (PET) columns (40 cm long and 5 cm in diameter). The experiment involved five sets of disturbed soil columns: (i) soil (200 g), (ii) soil (194 g) + OP (6 g), (iii) soil (194 g) + SG (6 g), (iv) soil (194 g) + GP (6 g), and (v) soil (194 g) + GW (6 g). The column was packed, and then air was removed using 0.01 M CaCl_2_. At last, the excess water was drawn off by gravity. Then, 0.6 mL of an acetonitrile/water (50/50 *v*/*v*) solution containing 100 μg mL^−1^ of each herbicide (spiking level = 0.5 μg g^−1^) was added to the top of each column. Twenty-four hours after the addition of the herbicides, the compounds were leached using 500 mL of 0.01 M CaCl_2_ that was added with a peristaltic pump. Eight leachates (50 mL × 6 and 100 mL × 2) were collected quantitatively over eight days and then filtered through a 0.22 μm nylon membrane filter. The columns were finally opened, and the soil was separated into two segments that were the same length. To prevent aqueous hydrolysis, the leachates were subjected to daily extraction of herbicide residues. The experiments were performed three times and shielded from direct light.

### 2.4. Analytical Determinations

A total of 10 g of dried soil samples was extracted with 15 mL of acetonitrile/water (2:1) via sonication. This process was carried out using a 200 W sonic dismembrator (Dr. Hielscher GmbH, Stahnsdorf, Germany). Following this extraction, a salting-out step was performed by adding 2 g of NaCl. The samples were then centrifuged for 10 min at 4000 rpm. Water samples were transferred directly to chromatographic vials after being filtered through 0.2 μm nylon filters. Herbicide residue determination was performed using a Waters e2695 separation module High Performance Liquid Chromatography (HPLC) system (Waters Corporation, Milford, CT, USA). The system was equipped with a quaternary pump, autosampler, and a Waters 2998 Photodiode Array Detector (PDA) (Waters Corporation, Milford, CT, USA). The information was handled using Empower 3 software from Waters. The separation was performed using a Phenomenex Kinetex XB-C18 (100 mm × 4.6 mm × 5 μm) analytical column (Madrid, Spain). A gradient mode was used for the mobile phase, which consisted of acetonitrile (solvent A) and water (solvent B). The temperature of the column oven was set to 25 °C. The following gradient was used: 30% A for one minute, increasing linearly to 90% A over nine minutes, holding for one minute, and decreasing to 30% A over two minutes to allow equilibration prior to the next injection (five minutes). The flow rate was 0.5 mL min^−1^, and the injection volume was 30 μL. We based the confirmation on retention times and recovery wavelengths (190–400 nm). The wavelengths at which the detection occurred were 247 and 249 nm for MB and CB, respectively.

The dissolved organic carbon (DOC) content was determined using a Multi N/C 3100 TOC Analyzer (Analytik Jena AG, Jena, Germany). Prior to analysis, the samples were filtered through a 0.45 mm nylon syringe filter to remove particulate organic carbon.

### 2.5. Statistical Analysis

Curve fitting was performed using SigmaPlot version 15.0 statistical software (Systat Software, Inc., San Jose, CA, USA). Standard deviation was used to assess variability among replicates. The Tukey post hoc test (*p* ≤ 0.05) was then used to determine significant differences in means with IBM SPSS Statistics version 29.0 (Armonk, NY, USA).

## 3. Results and Discussion

### 3.1. Adsorption of Herbicides in Amended and Unamended Soils

Following the experimental protocol specified in Section 2.3, Table 4 shows the experimental values found for the *K*_OC_ values in unamended and amended soils. As can be seen, adding the agro-industrial waste significantly increases the *K*_OC_ values in all cases (*p* < 0.05), especially in the soil amended with gazpacho waste and grape pomace. Values of log *K*_OC_ less than two indicate limited soil retention capacity, which can lead to leaching—in our case, these values are between two and four. This indicates a certain possibility of leaching. As stated by Blondel et al. [47], it seems that *K*_d_ is elevated when there are two halogen atoms on the phenyl group as opposed to just one as well as for compounds with no halogen atoms on the phenyl ring or one halogen atom.

Despite the fact that modern synthetic chemicals are more polar and soluble in water than their predecessors, organic matter remains the most important soil constituent influencing pesticide sorption, although other soil properties, such as the type and quantity of clay, can also affect sorption [9]. Since the pK_a_ of MB is 12.0 and the pH of the soil is 7.9, it will predominantly exist in its neutral (non-ionized) form in the soil and, by extension, CB, with a similarly high pK_a_. Neutral molecules tend to interact more strongly with SOM through van der Waals forces and hydrogen bonds, and less so with the surface charges of clay minerals. Therefore, the predominance of MB and CB in their neutral forms means their adsorption will be mainly influenced by OM and, to a lesser extent, the properties of clays. Navarro et al. [18] reported an increase in the adsorption of different phenylurea herbicides in soils with a high OM content (endoleptic phaeozem) compared to soils with a low OM content (hypercalcic calcisol). Pérez-Lucas et al. [20] have also reported an increase in the adsorption of isoproturon and chlortoluron in soils amended with agro-forestry, agro-industrial, and animal manure. Additionally, other authors have demonstrated that adding composted sheep manure and spent coffee grounds to clay loam soil (OM = 0.22%) significantly increases the sorption of ten different pesticides and, consequently, decreases their leaching potential [14]. Similarly, Yavarl et al. [25] demonstrated that adding biochar (a carbon-rich biosorbent) can stabilize polar herbicides in soil and potentially reduce their leaching. Similar results were obtained by Herrero-Hernández et al. [26] when evaluating the effect of applying spent mushroom substrate to soil on the adsorption, dissipation, and mobility of two fluopyram and tebuconazole (fungicides) in vineyard soils.

To limit the effect of exogenous organic matter on the compound’s effectiveness, it is necessary to keep the organic matter between the root zone, where the compound is needed, and the water table, where it is unwanted. However, enhancing the soil’s sorptive capacity to reduce the concentration of a herbicide in the soil solution makes the compound less available to weeds within the crop [48].

### 3.2. Dissipation and Persistence of Herbicides in Amended and Unamended Soils

A pesticide’s ability to maintain its molecular integrity, as well as its chemical, physical, and functional characteristics, in soil is commonly defined as persistence. Persistence is typically evaluated using its half-life (t_1/2_) or disappearance time (DT_50_) in SFO kinetics, which is the time required for the quantity of the pesticide in the soil to decrease by half. Table 5 shows the statistical parameters obtained for pesticide degradation following the SFO model. Based on the results obtained, the SFO model can adequately explain the degradation of both herbicides in both amended and unamended soils. This assertion is further substantiated by the calculated coefficients of determination (R ≥ 0.95), which surpass the threshold (R ≥ 0.7) proposed by OECD [45]. In this model, the rate of change in pesticide concentration (d*C*/d*t*) is directly proportional to the remaining soil concentration at any given time. Furthermore, *S*y/x values are no greater than 0.11, and *C*_0_ is nearly 1 in all cases. According to Gavrilescu’s [49] classification, pesticides can be categorized based on their soil persistence. Depending on whether the time is less than 30 days, between 30 and 110 days, or greater than 110 days, they can be cataloged as non-persistent, moderately persistent, or persistent, respectively. Consequently, MB exhibited medium persistence in unamended soil (S), yet it could be classified as non-persistent in soils treated with agro-industrial waste. Conversely, CB appears moderately persistent in all cases except in soil amended with orange peel (S + OP), where its DT_50_ is 18 days (non-persistent). Research shows that orange peel contains 23% sugar, 22% cellulose, 25% pectins, and 11% hemicellulose [50]. Contradictory effects of OM on the degradation of pesticides in soil have been observed [7,12]. On the one hand, OM favors the disappearance of herbicides in the soil during the initial stages by promoting biodegradation. On the other hand, OM increases the likelihood that herbicide residues will be sorbed to humic substances during the persistence phase, resulting in bound residues.

### 3.3. Leaching of Herbicides Through the Soil Columns

#### 3.3.1. Relative and Cumulative Breakthrough Curves (BTCs) of Herbicides

**Figure 1:**
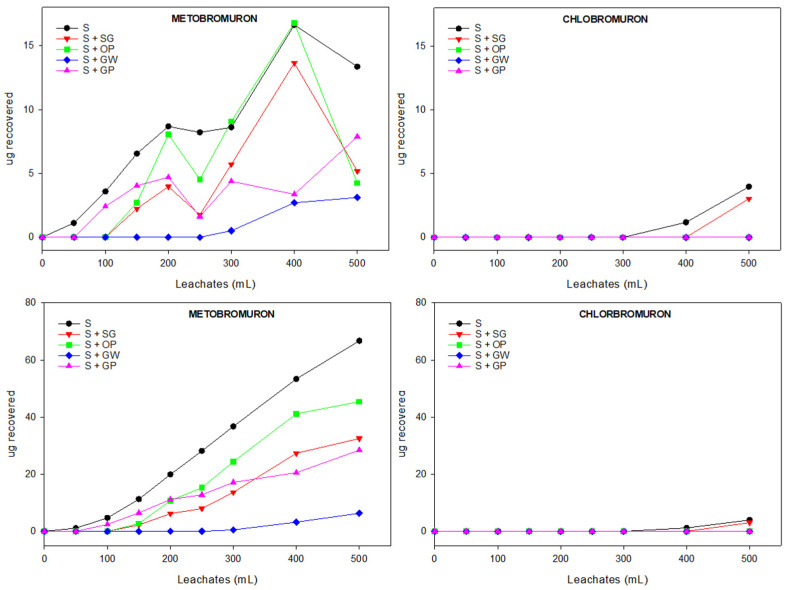
Breakthrough curves showing the relative (**top**) and cumulative (**bottom**) leaching of herbicides through amended and unamended soil columns.

Figure 1 shows the relative and cumulative BTCs of both herbicides in unamended and amended soils. The BTCs of herbicides in soil show asymmetry with tailing for both amended and unamended soils, which demonstrates their interaction with soil colloids, although this interaction occurs to different degrees. Pesticides often leach from soils asymmetrically, a behavior that has been attributed to non-equilibrium sorption. Time-dependent interactions between pesticides and soil colloids, such as clays and organic fractions, are the reason for this [51].

For MB, leaching begins quickly after 50 mL are passed and increases progressively up to 400 mL of leachates are recovered in unamended soil (S). A similar pattern is observed in soils amended with orange peel (S + OP) and spent brewers’ grains (S + SG), although in these cases, leaching begins after 100 mL. For soils amended with grape pomace (S + GP) and gazpacho waste (S + GW), leaching is significantly decreased, especially in the latter. This behavior can be explained by the higher values of Log *K*_OC_ obtained for MB in the soils amended with GP and GW. For CB, leaching only occurs in unamended soil (S) and in soil amended with spent brewers’ grains (S + SG), though the process begins after 400 mL was recovered from the columns. MB has higher water solubility and lower log *K*_OW_ and log *K*_OC_ values than CB (Table 1) due to the chloride atom present in the latter. This could explain the different behavior. Similar results were obtained by Fenoll et al. [52]. They demonstrated that adding agro-industrial wastes (coir and spent coffee grounds) and composted organic wastes (pine bark and sheep manure) added at a rate of 10% (*w*/*w*) increased the sorption of 14 substituted phenylurea herbicides, reducing their mobility in the soil and decreasing their leaching.

**Figure 2:**
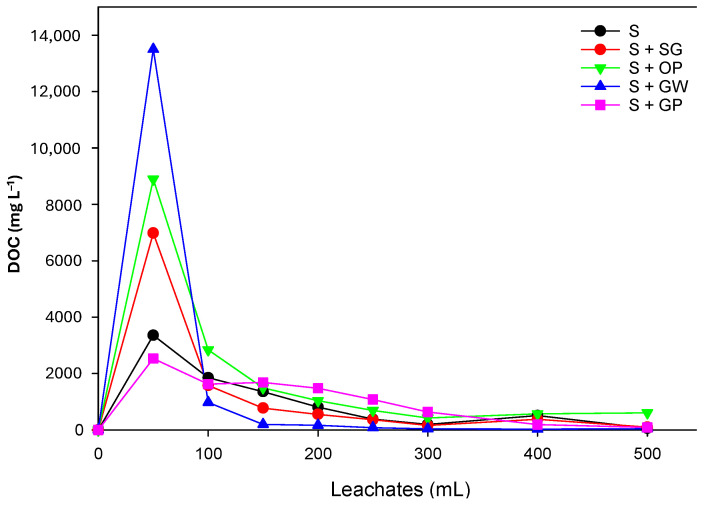
Concentration monitored of DOC in leachates in amended and unamended soils.

An increase in dissolved organic matter (DOM))is always induced by amending the soil [53], and the herbicides interact with DOM. Figure 2 shows the concentration of DOM determined in leachates. In all cases, the maximum concentration was observed in the first leachate (50 mL). The presence of DOM in earlier leachates, which are amber in color, could enhance the water solubility of herbicides through associations with DOM, making it easier for them to be initially leached. Sometimes, the presence of competing herbicides, like diuron (phenylurea), and DOM for sorption sites, rather than interactions in solution, can lead to increased pesticide leaching [54]. However, it is unclear to what extent the DOM is involved in the transport process. The movement of herbicides through the soil may increase if there is a significant interaction between the DOM and insoluble organic matter. Consequently, the concentration of DOM would influence the amount of herbicides in the leaching process.

#### 3.3.2. Distribution of Herbicides from Soil and Leachates

**Figure 3:**
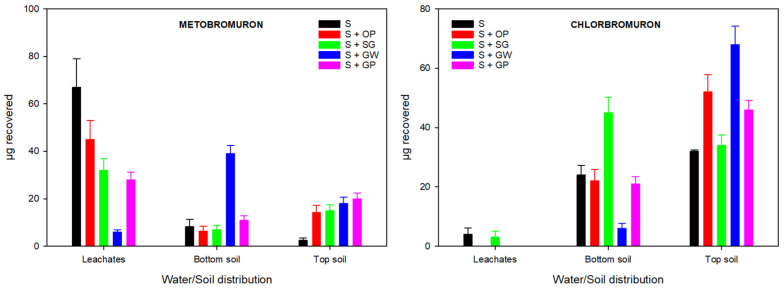
Distribution of herbicides in soil and total leachates in unamended and amended soils.

Figure 3 shows the distribution of herbicides added to unamended and amended soil columns from soil layers and leachates.

MB was found in all leachates, though at different levels. In unamended soil, 65% of the initial mass added to each column was recovered from the total leachate. Meanwhile, the mass recovered from amended soils ranged from 6% (S + GW) to 44% (S + OP). CB was only recovered in unamended soil (4%) and in soil amended with SG (3%). Consequently, CB was retained in the soil layers, especially the topsoil, at a higher proportion than MB, as can clearly be observed. Pérez-Lucas et al. [21] have demonstrated that other phenylurea herbicides, such as chlorotoluron and isoproturon, are highly leachable in unamended loam soil (OM = 1.1%), which reduces their leaching potential in soils amended with agroforestry, agro-industrial, and manure waste due to the increased adsorption on amended soils. Other authors have found that using different organic amendments, such as seed coats, chaff, wheat residues, olive mill waste, sewage sludge, grape pomace, spent mushroom compost, pine bark, composted sheep manure, spent coffee grounds, coir, and vermicomposted agro-industrial waste, significantly decreases the leaching rates of various pesticides, including phenylurea and triazine herbicides [16,17,51,52,53,54,55].

#### 3.3.3. Leaching Index Screening

Eight leaching screening indexes (GUS, Hornsby, LIN, LIX, LEACH, MLEACH, GLI, and ELI), which were extracted from the scientific literature, have been used to compare the leaching behavior of herbicides. The values of *K*_OC_, *k*, and t_1__/2_ obtained under our experimental conditions were used to calculate those indices. ELI was estimated using the amounts of herbicides that were recovered from the leachates. The indices studied and their interpretation criteria are shown in Table 6.

Table 7 shows the values of the calculated indices in both amended and unamended soils. As can be seen, all indices except GLI categorize MB as mobile or very mobile in unamended soil. For amended soils, the indices classify this herbicide as transitioning to mobile, except for LIX and GLI, which categorize it as immobile. For CB, some indices (GUS, LIX, LEACH, MLEACH, and Hornsby) classify this compound as a medium-to-high leacher in unamended soil, while GLI and ELI classify it as having low mobility. In amended soils, the leaching potential decreases because most of the indices catalog CB as immobile/transition.

## 4. Conclusions

Although pesticides help farmers produce high-quality food, they pose unavoidable risks due to their negative environmental impact through processes such as spray drift, runoff, and leaching. In southeastern Spain, adding organic amendments to the soil is a common practice used to improve soil health. Additionally, they significantly influence the sorption, leaching, and bioavailability of pesticides. The leaching potential of the studied herbicides (metobromuron and chlorbromuron) decreased due to the increased adsorption capacity of soil amended with agro-industrial waste products (orange peel, beer bagasse, grape pomace, and gazpacho waste). This decreases the bioavailability of pesticides. Therefore, using organic amendments is a valuable strategy for reducing the risk of crop and groundwater pollution from pesticides while contributing to the circular economy. Using screening models or leaching indices, such as GUS, LIX, LEACH, M-LEACH, LIN, GLI, HI, and ELI, among others, is a valuable tool for predicting the vulnerability of groundwater to herbicide pollution, although the reliability of these indices is not always in agreement.

Further investigations under field conditions are needed to compare the results obtained in our experiment with those obtained using other rainfall patterns. This includes studying transformation products during risk assessments and monitoring groundwater. Therefore, it is crucial to identify possible strategies to present to national and local governments, water advisory boards, and farmers. The goal is to reduce the presence of herbicides in groundwater to ultimately lead to cleaner drinking water.

## Figures and Tables

**Table 1:** Physical-chemical properties of the herbicides used in this study [41,42].

Herbicide	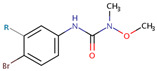	Molecular Formula	Molecular Mass	log *K*_OW_	S_W_^a^	log *K*_OC_^b^			GUS ^c^
						Exp.	MCI	log *K*_OW_	
Chlorbromuron	R = Cl	C_9_H_10_BrClN_2_O_2_	293.5	3.1	35	2.70	2.53	2.58	2.07
Metobromuron	R = H	C_9_H_11_BrN_2_O_2_	259.1	2.4	330	2.10	2.32	2.19	2.42

^a^ Water solubility (mg L^−1^); ^b^ in parentheses, the experimental and estimated values (Molecular Connectivity Index and log *K*_OW_) taken from KOCWIN ™ (EPI Suite v 4.10) are shown; ^c^ Groundwater Ubiquity Score Index.

**Table 2:** Physical-chemical properties of the soil used.

Sand	Silt	Clay	pH	EC (dS m^−1^)	OM (%)	OC (%)	TN (%)	C/N	CaCO_3_ (%)	CEC (cmol^+^ kg^−1^)
10.1%	58.4	31.5%	7.9	0.24	0.6	0.35	0.08	8	40.3	10.7

**Table 3:** Main physicochemical properties of the agro-industrial wastes used as organic soil amendments (n = 3).

**Waste**	**pH (1:5)**	**EC (ds m^−1^) (1:5)**	**% OM**	**% OC (OC_H_)**	**% N**	**C/N**	**% FA**	**% HA**	**% Ashes**
SG	4.53	1.17	79.63	46.19 (1.8)	5.14	8.99	3.20	1.00	3.81
GP	3.56	2.77	80.45	46.67 (1.3)	2.03	22.99	2.10	2.45	6.56
GW	5.17	2.52	85.23	49.44 (2.9)	2.46	20.10	1.93	1.21	4.20
OP	3.14	1.42	73.61	42.7 (2.9)	1.08	39.54	4.03	0.65	3.78
	**Anions**				**Cations**				
	**F^−^**	**Cl^−^**	**NO_3_^−^**	**PO_4_^−^**	**SO_4_^−^**	**K**	**Ca**	**Mg**	**Na**
SG	3446	434	40	5330	1632	0.09	0.17	0.21	0.01
GP	871	93	378	5162	45138	0.19	0.43	0.09	0.01
GW	759	3956	1362	12398	4762	0.36	0.18	0.21	0.05
OP	4504	2346	2550	4562	5142	0.05	0.85	0.08	0.05

EC: electrical conductivity, OM: organic matter, OC: organic carbon, OC_H_: water-soluble organic carbon, N: nitrogen, C/N: carbon/nitrogen ratio, FA: fulvic acid, HA: humic acid; anions (mg kg^−1^, extract 1:5); cations (g 100 g^−1^).

**Table 4:** Log *K*_OC_ values calculated for herbicides in unamended and amended soils.

Soil	Log *K*_OC_	
	Metobromuron	Chlorbromuron
S	1.9	2.5
S + OP	2.1	2.6
S + SG	2.2	2.7
S + GW	2.4	3.0
S + GP	2.4	2.9

**Table 5:** Parameters obtained (n = 3) according to SFO kinetics for the dissipation of the studied herbicides in amended and unamended soils.

Herbicide	Soil	Parameter					
		R^2^	*C*_0_	*K* (d^−1^)	*S*_y/x_	DT_50_ (d)	DT_90_ (d)
Metobromuron	S	0.974 ***	0.96	0.0124	0.04	56	186
	S + OP	0.962 **	1.07	0.0475	0.10	15	48
	S + SG	0.989 ***	1.03	0.0650	0.05	11	35
	S + GW	0.995 ***	1.00	0.0448	0.03	15	51
	S + GP	0.951 **	1.07	0.0425	0.11	16	54
Chlorbromuron	S	0.979 ***	0.97	0.0073	0.02	95	315
	S + OP	0.966 ***	1.03	0.0097	0.04	71	237
	S + SG	0.972 ***	0.95	0.0390	0.06	18	59
	S + GW	0.959 ***	1.05	0.0130	0.05	53	177
	S + GP	0.978 ***	0.99	0.0208	0.05	33	111

** (*p* < 0.01); *** (*p* < 0.001); *S*_y/x_: Standard error of estimation.

**Table 6:** Indices used to assess herbicide leaching potential.

Index	Equation	Criteria
GUS [56]	$GUS=\left[4-\log(K_{OC})\right]*\log(t_{½})$	GUS > 2.8: leachable; GUS = 1.8–2.8: transition; GUS < 1.8: non-leachable
Hornsby Index [57]	$HI=\left(\frac{K_{OC}}{t_{½}}\right)*10$	HI ≤ 10: high; HI ≥ 2000: low
LIN [58]	$LIN=-0.531\;logK_{OW}+0.518\;logS_{w}-0.495\;\log K_{OC}-0.023\;\log V_{p}-0.452\;\log K_{H}$	Comparison (lower values, lower leaching potential)
LIX [59]	$LIX=\exp(-k*K_{OC})$	LIX = 1: high leachable; LIX = 0.1–1: leachable; LIX = 0–0.1: transition; LIX = 0: non-leachable
LEACH [60]	$LEACH=\frac{S_{w}*t_{½}}{V_{p}*K_{OC}}$	Comparison (lower values, lower leaching potential)
M. LEACH [61]	$M.LEACH=\frac{S_{w}*t_{½}}{K_{OC}}$	Comparison (lower values, lower leaching potential)
GLI [61]	GLI=0.579 LIN+0.558 GUS+0.595 M.LEACH	GLI > 1: high; GLI = −0.5–1: medium; GLI < −0.5: low
ELI [21]	$ELI=\frac{M_{LIX}*V_{W}*Fmap}{M_{S}}$	ELI ≤ 0.1: Immobile; 0.1 > ELI ≤ 0.6: Transition; 0.6 > ELI ≤ 1.5: Mobile, 1.5 > ELI ≤ 2: Very mobile.

t_1/2_: half-life (days); Sw: water solubility (mg L^−1^); V_P_: vapor pressure (mm Hg); K_H_: Henry Law constant; K_ow_: octanol/water partition coefficient; K_oc_: organic carbon normalized soil sorption coefficient (mL g^−1^) organic carbon); M_LIX_: total recovered mass (μg)/added mass (μg), V_W_ (mL): total volume in leachates according to the mean annual precipitation in a specific place; M_S_ (g): mass of soil used; F: correction factor bearing in mind the mean annual precipitation in a specific place.

**Table 7:** Leaching indices calculated for metobromuron and chlotoluron in amended and unamended soils.

Soil	Index							
	GUS	LIX	LEACH	MLEACH	LIN	GLI	HORNSBY	ELI
Metobromuron								
S	3.67 ^4^	0.37 ^4^	1606 ^4^	231 ^3^	0.84 ^3^	140 ^1^	14 ^3^	1.31 ^4^
S + OP	2.23 ^2^	0.00 ^1^	271 ^3^	39.1 ^2^	0.74 ^3^	24.9 ^1^	84 ^3^	0.89 ^3^
S + SG	1.87 ^2^	0.00 ^1^	158 ^2^	23.2 ^2^	0.70 ^3^	15.0 ^1^	144 ^3^	0.64 ^3^
S + GW	1.88 ^2^	0.00 ^1^	136 ^2^	19.6 ^2^	0.60 ^3^	13.1 ^1^	167 ^3^	0.12 ^2^
S + GP	1.93 ^2^	0.00 ^1^	145 ^2^	20.9 ^2^	0.60 ^3^	13.9 ^1^	157 ^3^	0.56 ^2^
Chlorbromuron								
S	2.97 ^4^	0.10 ^4^	198 ^3^	10.5 ^3^	−0.43 ^2^	7.7 ^1^	33 ^3^	0.08 ^1^
S + OP	2.59 ^2^	0.02 ^2^	113 ^3^	6.2 ^3^	−0.48 ^2^	4.9 ^1^	56 ^3^	0.00 ^1^
S + SG	1.63 ^2^	0.00 ^1^	23 ^2^	1.3 ^2^	−0.53 ^2^	1.2 ^1^	278 ^3^	0.06 ^1^
S + GW	1.72 ^2^	0.00 ^1^	35 ^2^	1.9 ^2^	−0.68 ^2^	1.7 ^1^	188 ^3^	0.00 ^1^
S + GP	1.67 ^2^	0.00 ^1^	27 ^2^	1.5 ^2^	−0.63 ^2^	1.4 ^1^	240 ^3^	0.00 ^1^

^1^ Immobile, ^2^ transition, ^3^ mobile, ^4^ very mobile.

## Data Availability

The original contributions presented in this study are included in the article. Further inquiries can be directed to the corresponding author(s).

## Abbreviations

The following abbreviations are used in this manuscript:

BTCs	Breakthrough curves
CB	Chlorbromuron
DOC	Dissolved organic carbon
DT	Disappearance time
EC	European Commission
FOCUS	(Forum for the Co-ordination of pesticide fate models and their Use)
GP	Grape pomace
GW	Gazpacho wastes
MB	Metobromuron
OC	Organic carbon
OECD	Organization for Economic Cooperation and Development
OM	Organic matter
OP	Orange peel
SFO	Single first order
SG	Spent grains
SOM	Soil organic matter
US EPA	United States Environmental Protection Agency
